# Results of an interventional HIV testing programme in the context of a mpox (formerly monkeypox) vaccination campaign in Latium Region, Italy, August to October 2022

**DOI:** 10.2807/1560-7917.ES.2022.27.48.2200890

**Published:** 2022-12-01

**Authors:** Silvia Pittalis, Valentina Mazzotta, Nicoletta Orchi, Isabella Abbate, Roberta Gagliardini, Elisabetta Gennaro, Augusto Faticoni, Pierluca Piselli, Gabriella Rozera, Stefania Cicalini, Fabrizio Maggi, Enrico Girardi, Francesco Vaia, Andrea Antinori, Vincenzo Puro

**Affiliations:** 1 National Institute for Infectious Diseases Lazzaro Spallanzani – IRCCS, Rome, Italy

**Keywords:** sexually transmitted infections, HIV infection, monkeypox virus (MPOXV), public health policy

## Abstract

HIV testing was offered to 2,185 people receiving mpox (formerly monkeypox) vaccination, who reported not being HIV positive. Among them 390 were current PrEP users, and 131 had taken PrEP in the past. Of 958 individuals consenting testing, six were newly diagnosed with HIV. Two patients had symptomatic primary HIV infection. None of the six patients had ever taken PrEP. Mpox vaccination represents an important opportunity for HIV testing and counselling about risk reduction and PrEP.

Mpox (formerly monkeypox) is a viral zoonosis, which can also spread between people through close physical contact [1]. While the disease is endemic to parts of Africa, a mpox outbreak affecting several countries outside this continent was reported by the World Health Organization (WHO) in May 2022 [1]. On 23 July 2022, due to the large international spread of the outbreak, WHO declared it a Public Health Emergency of International Concern [2], and shortly after issued interim guidance on immunisation with available vaccines [3]. Because, currently, mpox transmission is mainly occurring in defined interconnected sexual networks involving men who have sex with men (MSM), and risk behaviours for mpox overlap with those for sexually transmitted infections (STIs), most notably HIV [4,5], mpox vaccination campaigns present a potential opportunity to test for HIV and promote pre-exposure prophylaxis (PrEP) [6].

## Mpox vaccination campaign

On 8 August 2022, the National Institute for Infectious Diseases ‘Lazzaro Spallanzani’ in Rome, Italy, initiated a vaccination campaign for people at high-risk for mpox in the Lazio Region. Our Institute is the regional reference centre for diagnosis and care of HIV/AIDS and includes in- and out-patient clinics for people living with HIV (PLWH) and a Voluntary Counseling and Testing Site (VCTS), also in charge of providing post-exposure prophylaxis and PrEP. According to the recommendations of the Italian Ministry of Health, the target population for mpox vaccination has been identified as gay, transgender, bisexual, and MSM individuals reporting more than one sexual partner in the last 3 months, or participation in group sex events, or sexual encounters in clubs/cruising/saunas, or recent STI, or practicing chemsex. The non-replicating modified vaccinia Ankara (MVA-BN; Bavarian Nordic) was made available [7].

## HIV testing during the vaccination campaign

When individuals presented to attend the vaccination campaign, data were first collected to confirm that the criteria for vaccination were fulfilled. Risk reduction counselling was offered to all people undergoing vaccination, and testing for HIV was offered to those reporting not being PLWH. A signed consent was required to be HIV tested.

The HIV counsellor recorded in a patient chart the personal information obtained on a confidential basis from individuals consenting to be tested. This information included socio-demographic characteristics, behavioural data, and a brief medical history, including information on other STIs.

Blood samples were tested for HIV using a fourth-generation test for HIV-1/2 antibodies (ARCHITECT HIV** **Ag/Ab Combo Assay, Abbott), and reactive samples (i.e. index value > 1) were confirmed by immunoblot (Geenius HIV** **1/2 Confirmatory Assay, Bio-Rad), with results available within 72 hours from blood drawing. A subset of attenders asked for a rapid test. These attenders were tested on blood obtained by fingerstick using a fourth-generation rapid test that detects both HIV-1/2 antibodies and free HIV-1 p24 antigen (DETERMINE HIV-1/2 AG/AB COMBO, Abbott), confirmed by a conventional blood test when positive.

Individuals not consenting to HIV testing or testing HIV negative were invited to attend the VCTS for risk reduction counselling and for considering if PrEP was appropriate for them. Those testing HIV positive were promptly referred to our HIV outpatient clinic to start antiretroviral therapy. Epidemiological and clinical data (CD4^+^ cell count, HIV viral load, AIDS diagnosis) were routinely collected.

For the purpose of this communication, we reviewed charts and analysed routinely collected data, which were anonymised by coding all personal identifiers.

## Diagnoses of HIV at the time of vaccination

Up to 28 October 2022, 3,076 individuals received at least one dose of MVA-BN vaccine. Overall, 29% (n = 891) of them were PLWH, all receiving antiretroviral therapy. Of the 2,185 who reported not being HIV positive, 390 were current PrEP users, and 131 had taken PrEP in the past but were not currently taking it.

A total of 1,227 (56%) individuals did not consent to be tested, including 300 current PrEP users. Of the remaining 958 (44%) individuals who consented to an HIV test, six (0.63%; 95% confidence interval (CI): 0.13%–1.13%) tested positive (Table). Notably, none of the six patients had ever taken PrEP.

**Table:** Characteristics of individuals newly diagnosed with HIV infection at time of mpox vaccination, National Institute for Infectious Diseases, Rome, Italy, 8 August–28 October 2022 (n = 6)

Characteristics	Patient 1	Patient 2	Patient 3	Patient 4	Patient 5	Patient 6
Age range in years	30–39	20–29	40–49	40–49	30–39	40–49
Practice of chemsex	No	No	No	No	No	No
Previous STIs	Urethral gonorrhoea	Urethral gonorrhoea	Secondary syphilis; acute hepatitis A	Acute hepatitis B; latent syphilis	None	None^a^
Duration since last negative HIV test	< 1 year	None	ca 3 years	< 1 year	ca 5 years	ca 2 years
Previous PEP, if so year	Yes, 2015	No	No	No	No	No
Lymphocytes CD4^+^ count in cells/mL	720	413	181	743	672	383
HIV viral load in copies/mL, (detection range 30–10,000,000)	2,155	> 10,000,000	133,544	27,519	14,214	641,632
Type of infection and estimate of time of infection [8]	Recent infection (< 1 year)	Symptomatic PHI (< 1 month)	Advanced, late diagnosed (> 1 year)	Symptomatic PHI (> 1 month < 6 month)	Chronic infection (> 1 year)	Chronic infection (> 1 year)
Symptoms/signs before the diagnosis	Not reported	Fever, myalgia and diarrhoea up to 6 days before	Not reported	Diffuse macular cutaneous rash, swollen neck lymph nodes, feeling of fever and fatigue ca 3 months before	Not reported	Not reported

PEP: post-exposure prophylaxis for HIV; PHI: primary HIV infection; STIs: sexually transmitted infections.

^a^ Latent syphilis at the time of HIV diagnosis.

Patients 2 and 4 were both diagnosed with symptomatic primary HIV infection (PHI) [8], while Patient 3 received a HIV diagnosis at an advanced stage of infection.

Patient 2 was a man in his 20s who had never been prior tested for HIV; he reported a recent diagnosis of urethral gonorrhoea and a 4-day episode of fever, myalgia and diarrhoea up to 6** **days before the HIV test. The test results suggested a PHI (Fiebig stage IV) [8], with a positive fourth-generation Ab/Ag screening test and an indeterminate immunoblot (only gp41 reactivity); antigen p24 concentration was > 400pg/µL (VIDAS HIV P24, bioMérieux) and plasma HIV-1 RNA concentration > 10,000,000 copies/mL (Aptima HIV-1 Quant Dx assay, Hologic); CD4^+^ count was 413 cells/µL (36.8%).

The HIV test results of Patient 4, who was in his 40s, also suggested a recent HIV acquisition, with an immunoblot positivity lacking p31 reactivity (Fiebig stage V) [8] and an anti-HIV IgG antibody avidity index < 0.80. He reported a last HIV negative test in early 2022, as well as having had acute hepatitis B and latent syphilis diagnoses in the past. Appearance of a diffuse macular cutaneous rash, feeling of fever and fatigue were reported to have occurred ca 1 month before the current HIV test. His CD4^+^ count was 743 cells/µL (27,4%), and plasma HIV-1 RNA concentration 27,519 copies/mL.

Patient 3, equally in his 40s, received a late HIV diagnosis. He was asymptomatic and reported a last HIV negative test around 3 years before, as well as diagnoses, many years prior, of secondary syphilis and of acute hepatitis A. The lymphocyte CD4^+^ count for this patient was 181 cells/µL, and the HIV-RNA concentration 133,544 copies/mL.

Patient 1, who reported a last negative HIV test less than 1 year prior (late 2021) had a recent infection; he reported HIV PEP 7 years before, and treatment for urethral gonorrhoea in the past. Patients 5 and 6 had chronic infection. The last HIV negative test reported by Patient 5 dated back more than 5 years; CD4^+^ count was 672 cells/µL (39%) and HIV RNA 14,214 copies/mL. Patient 6 reported a last HIV negative test 30 months before; his CD4^+^ count was 383 cells/mL and HIV RNA concentration 641,632 copies/mL; he was diagnosed with latent syphilis at the time of the HIV diagnosis.

## Discussion

Our data confirm the importance of taking every available opportunity to offer HIV testing, particularly to high-risk populations, to improve early diagnosis and treatment and to contribute to achieving the WHO 95–95–95 goal by 2030 [8-10].

In our study, the consent rate to HIV testing was 44% overall, reaching 50% if we do not consider in the denominator those 300 individuals who declined the test because of being on PrEP and likely recently HIV tested. This finding cannot be considered satisfying and confirms the need for further efforts to overcome barriers to HIV testing.

Of note, only 390 individuals among our population targeted for mpox vaccination were currently taking PrEP, and additional 131 had been on PrEP in the past. Although a recall bias (i.e. individuals omitted the information) cannot be ruled out, the high proportion of individuals without HIV having high-risk behaviour and who are not on PrEP programmes represents an urgent public health challenge to address [11,12].

It is therefore necessary to investigate and try to break down the barriers to PrEP, to increase awareness about this strategy, also by involving the communities supporting people at high risk of HIV, and to implement targeted PrEP programmes, which are still lacking in Italy.

A potential limitation of our study is due to its retrospective nature, as a review of charts that were originally not designed to collect data for research, whose results could be affected by recall biases, and where some information is bound to be missing [13]. Indeed, in our study no data are available about reasons for not consenting to be tested, or for not being on PrEP, limiting the generalisability of some findings. 

## Conclusion

Our data show that the mpox vaccination campaign represents an important opportunity to take contact with people at high risk of HIV and other STIs. We recommend that everyone undergoing mpox vaccination should be offered HIV testing and, if they are negative, counselled about HIV/STI risk-reduction measures including PrEP.
